# Subthalamic Nucleus Deep Brain Stimulation in the Beta Frequency Range Boosts Cortical Beta Oscillations and Slows Down Movement

**DOI:** 10.1523/JNEUROSCI.1366-24.2024

**Published:** 2025-01-09

**Authors:** Lucy M. Werner, Alfons Schnitzler, Jan Hirschmann

**Affiliations:** Institute of Clinical Neuroscience and Medical Psychology, Medical Faculty and University Hospital Düsseldorf, Heinrich Heine University Düsseldorf, Düsseldorf 40225, Germany

**Keywords:** beta oscillations, deep brain stimulation, entrainment, magnetoencephalography, Parkinson’s disease

## Abstract

Recordings from Parkinson's disease (PD) patients show strong beta-band oscillations (13–35 Hz), which can be modulated by deep brain stimulation (DBS). While high-frequency DBS (>100 Hz) ameliorates motor symptoms and reduces beta activity in the basal ganglia and motor cortex, the effects of low-frequency DBS (<30 Hz) are less clear. Clarifying these effects is relevant for the debate about the role of beta oscillations in motor slowing, which might be causal or epiphenomenal. Here, we investigated how subthalamic nucleus (STN) beta-band DBS affects cortical beta oscillations and motor performance. We recorded the magnetoencephalogram of 14 PD patients (nine males) with DBS implants while on their usual medication. Following a baseline recording (DBS OFF), we applied bipolar DBS at beta frequencies (10, 16, 20, 26, and 30 Hz) via the left electrode in a cyclic fashion, turning stimulation on (5 s) and off (3 s) repeatedly. Cyclic stimulation was applied at rest and during right-hand finger tapping. In the baseline recording, we observed a negative correlation between the strength of hemispheric beta power lateralization and the tap rate. Importantly, beta-band DBS accentuated the lateralization and reduced the tap rate proportionally. The change in lateralization was specific to the alpha/beta range (8–26 Hz), outlasted stimulation, and did not depend on the stimulation frequency, suggesting a remote-induced response rather than entrainment. Our study demonstrates that cortical beta oscillations can be manipulated by STN beta-band DBS. This manipulation has consequences for motor performance, supporting a causal role of beta oscillations.

## Significance Statement

The slowing of movement in Parkinson's disease is known to be related to pathologically enhanced neural oscillations in the beta range (13–35 Hz). Whether these oscillations are the cause of motor slowing, however, remains debated. Here, we stimulated the brains of Parkinson’s patients in the beta range, using their implanted deep brain stimulator. Consistent with a causal effect, stimulation slowed down finger tapping. Additionally, the subcortical stimulation affected the hemispheric balance of beta oscillations in the remote motor cortices. The stronger the shift, the stronger the slowing. These results suggest that subcortical beta-band stimulation causes motor slowing by boosting motor cortical beta oscillations, indicating that beta oscillations might indeed be “a bad boy in Parkinson's disease.”

## Introduction

Parkinson's disease (PD) is a neurodegenerative disorder characterized by a spectrum of motor symptoms such as rigidity, bradykinesia, postural instability, and tremor (Jankovic, 2008). Electrophysiological recordings from PD patients commonly reveal pathologically enhanced oscillations in the beta-band (13–35 Hz), particularly within the subthalamic nucleus (STN) and other regions of the basal ganglia (Kuhn et al., 2006; Neumann et al., 2016). These neuronal oscillations are linked to motor symptoms (Brown et al., 2001; Cassidy et al., 2002; Neumann et al., 2016; van Wijk et al., 2016) and can be modulated by means of deep brain stimulation (DBS). For instance, DBS at 130 Hz has been shown to suppress beta oscillations in the STN (Meissner et al., 2005; Kühn et al., 2008; Eusebio et al., 2011) with the strength of this suppression correlating with the degree of motor improvement (Oswal et al., 2016). Accordingly, beta power suppression has been shown to be a valid predictor for bradykinesia improvement (Feldmann et al., 2022). High-frequency DBS has further been shown to suppress cortical beta oscillations in some studies (Abbasi et al., 2018; Muthuraman et al., 2021), but the behavioral correlates of these remote effects remain unclear.

Due to the well-established link between beta oscillations and bradykinesia, beta oscillations have been labeled “antikinetic” (Brown, 2003). This label suggests that elevated beta activity causes the slowing of movement in PD, touching on the longstanding debate on the mechanistic versus epiphenomenal role of oscillations (Neumann et al., 2016). Essential evidence for a mechanistic role might come from experiments that induce behavioral changes by modulating oscillations (Herrmann et al., 2016). Indeed, previous studies have demonstrated that STN stimulation at beta frequencies, as opposed to frequencies above 100 Hz, slows down movement (Chen et al., 2007; Eusebio et al., 2008). In addition, entrainment of cortical beta activity with transcranial alternating current stimulation resulted in a slowing of movement in healthy subjects (Pogosyan et al., 2009; Joundi et al., 2012).

In light of these observations, it seems possible that subthalamic DBS at beta frequencies leads to motor slowing by entraining or otherwise modulating beta oscillations in the motor cortex. Here, we addressed this possibility by applying unilateral beta-band DBS at rest and during finger tapping, while monitoring cortical oscillations by means of magnetoencephalography (MEG). We expected beta-band DBS to boost cortical beta oscillations, with the effect possibly outlasting stimulation. In accordance with previously reported effects (Fogelson et al., 2005; Chen et al., 2007; Eusebio et al., 2008; Pogosyan et al., 2009; Joundi et al., 2012), we further expected beta-band DBS to slow down movement.

## Materials and Methods

### Patients

**Figure 1.**
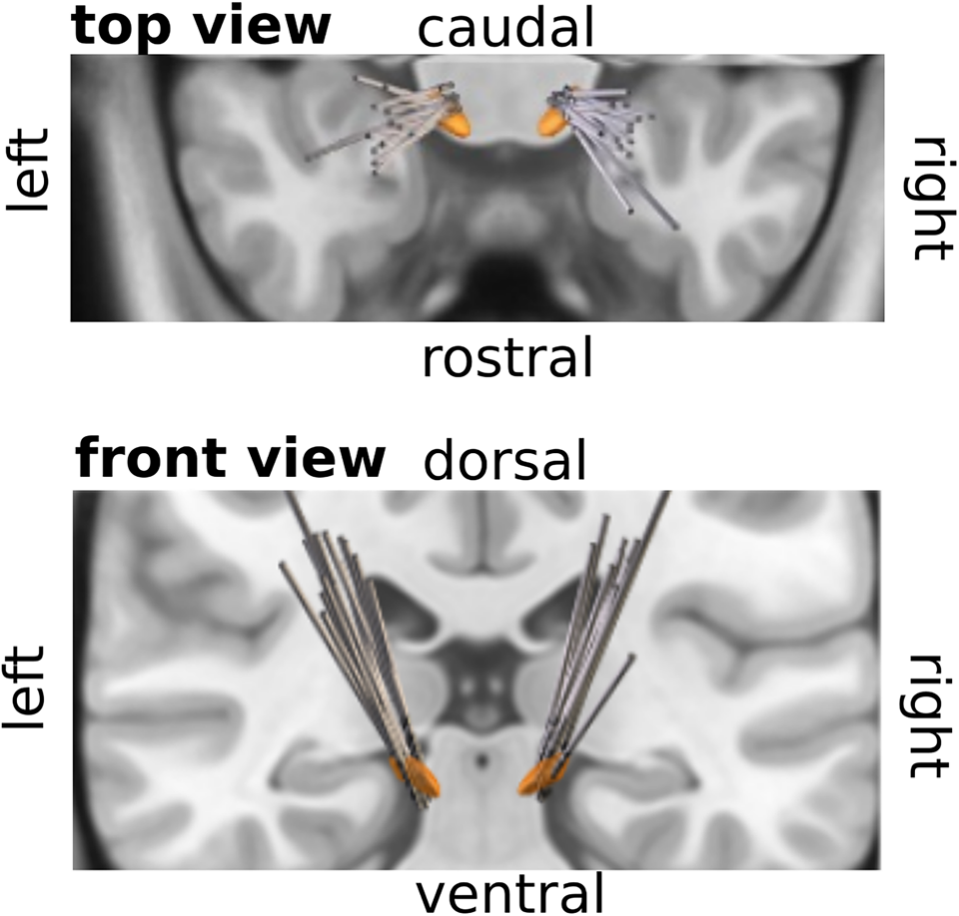
Reconstruction of electrode locations. The subthalamic nucleus is shown in orange.

Eighteen patients with idiopathic PD participated in the study. Patients were recruited at the Center for Movement Disorders and Neuromodulation, University Hospital Düsseldorf, during routine, inpatient, or outpatient visits. Four patients were excluded due to poor data quality or early experiment termination, caused by fatigue, resulting in a final sample of 14 patients (nine males, 63.50 ± 7.48 years, Table 1). All patients were implanted with an STN DBS system, and electrode localization, performed with Lead-DBS v3.1 (Horn and Kühn, 2015), confirmed the correct placement of all electrodes included in the study (Fig. 1). The medication schedule was not changed for this study. The study was carried out in accordance with the Declaration of Helsinki and was approved by the local ethics committee (study number 6233R). All patients provided written informed consent before enrollment.

**Table 1.** Patient demographic data and blocks acquired per subject

ID	Age	Sex	Motor subtype	DBS system	DBS frequency	MDS-UPDRS III Med OFF/Stim OFF	MDS-UPDRS III Med OFF/Stim ON
1	73	M	Tremor	Abbott Infinity	10, 16, 20, 26, 30 Hz	31	21
2	58	F	Mixed	Abbott Infinity	10, 16, 20, 26, 30 Hz	34	19
3	55	F	Mixed	Abbott Infinity	10, 30 Hz	41	29
4	72	F	Mixed	Abbott Infinity	10, 20, 30 Hz	25	NA
5	56	M	Tremor	Abbott Infinity	10, 16, 20, 26, 30 Hz	42	27
6	58	M	Tremor	Abbott Infinity	10, 16, 20, 26, 30 Hz	19	9
7	64	M	Tremor	Abbott Infinity	10, 16, 20, 26, 30 Hz	25	12
8	65	F	Mixed	Abbott Infinity	16, 30 Hz	NA	NA
9	72	M	Mixed	Abbott Infinity	10, 16, 20, 26 Hz	24	15
10	67	M	Mixed	Abbott Infinity	10, 16, 20, 26, 30 Hz	27	15
11	50	F	Tremor	Abbott Infinity	10, 16, 20, 26, 30 Hz	27	15
12	59	M	Tremor	Abbott Infinity	10, 16, 20, 26, 30 Hz	41	15
13	71	M	Tremor	Abbott Infinity	10, 16, 20, 26, 30 Hz	34	13
14	69	F	Mixed	Medtronic Percept PC	16, 26, 30 Hz	64	NA

NA, not available.

### DBS settings

Unilateral bipolar DBS was applied at various frequencies in the beta range (10, 16, 20, 26, and 30 Hz) using the left electrode in a cyclic fashion, such that the stimulation turned on (5 s) and off (3 s) repeatedly. The pulse amplitude was set to 3 mA. Unilateral DBS was chosen to facilitate comparisons between the stimulated and unstimulated hemisphere. Bipolar DBS was chosen to minimize interference from DBS artifacts during MEG recordings.

In case the patient was treated with monopolar DBS, the cathode was changed from the stimulator to the ring contact furthest apart from the active contact (the most dorsal contact usually). In case the patient's therapeutic stimulation was bipolar, the contact pair remained unchanged. The DBS settings were tested for side effects by a movement disorders neurologist before the recordings started.

### Electrode localization

DBS electrodes were localized using the advanced processing pipeline (Horn et al., 2019) in Lead-DBS v3.1 (lead-dbs.org; Horn and Kühn, 2015). In short, postoperative CT images were linearly coregistered to preoperative MRIs (T1 and T2) using advanced normalization tools (ANTs; stnava.github.io/ANTs/; Avants et al., 2011). Coregistrations were inspected and refined if needed. A brain shift correction step was applied as implemented in Lead-DBS. All preoperative volumes were used to estimate a precise multispectral normalization to ICBM 2009b NLIN asymmetric (“MNI”) space (Fonov et al., 2011) applying the ANTs SyN Diffeomorphic Mapping (Avants et al., 2008) using the preset “effective: low variance default + subcortical refinement.” DBS contacts were either manually reconstructed or automatically using the PaCER method (Husch et al., 2018). Atlas segmentations in this manuscript are defined by the DISTAL atlas (Ewert et al., 2018). Group visualizations were performed using the Lead group toolbox (Treu et al., 2020).

### Study design

The study had six experimental conditions: DBS OFF (baseline) and cyclic DBS at 10, 16, 20, 26, and 30 Hz. For each condition, we acquired one resting-state recording lasting 8 min, followed by a 3 min motor task (see below, Tapping task). Each session started with the baseline recording, followed by the remaining blocks in random order.

### MEG recordings

MEG recordings were conducted using a 306-channel, whole-head MEG system (VectorView, MEGIN) with a sampling rate of 2,000 Hz. Simultaneously, horizontal, and vertical eye movements, as well as muscle activity, were monitored via electrooculography and electromyography (EMG). EMG surface electrodes were attached to patients’ right and left forearms, referencing the muscle tendons at the wrist. Additionally, EMG surface electrodes were placed on the left chest to monitor cardiac activity. Further electrodes were positioned above the implanted stimulator and on the neck above the subcutaneous wire, facilitating the monitoring of the DBS artifact. The respective reference electrodes were positioned over the cervical vertebrae.

### Tapping task

**Figure 2.**
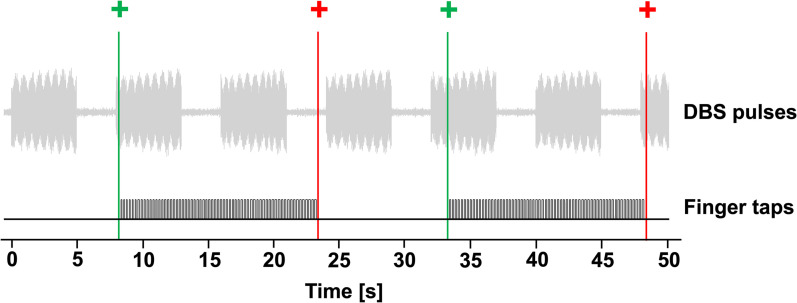
Cyclic deep brain stimulation in the finger tapping task. Top, DBS pulses, recorded in one magnetoencephalography channel, during 30 Hz DBS. The stimulation was turned on (5 s) and off (3 s) repeatedly. Bottom, Finger taps recorded simultaneously. Patients were instructed to begin tapping when a green fixation cross appeared and to stop when the cross turned red.

Patients were seated in the MEG shielded room, with a tap pad placed on a table in front of them, equipped with a light barrier. Visual stimulus presentation was controlled using Presentation (Neurobehavioral Systems). The task consisted of seven consecutive tapping blocks, each beginning with a 10 s display of a red fixation cross at the screen's center followed by a 15 s presentation of a green fixation cross. Patients were instructed to withhold any response during presentation of the red fixation cross and to keep their finger outside the light barrier. During presentation of the green fixation cross, they were instructed to tap as fast as possible, with the largest amplitude possible. Patients tapped with their right index finger only. Each block had a duration of 25 s, and the task lasted approximately 3 min. A visual representation of cyclic DBS in the tapping task is shown in Figure 2.

### Data analysis

#### Preprocessing

The data were preprocessed and analyzed using the MNE-Python library (Gramfort et al., 2014). We used temporal Signal Space Separation to mitigate interference originating from environmental magnetic field sources and DBS artifacts in particular (Taulu and Simola, 2006). Channels with poor signal quality and time segments containing muscle artifacts were excluded from further analysis after visual inspection. On average, we excluded 3.43 channels per participant (SD = 3.18). Data were segmented into epochs with a duration of ±3.5 s around the last pulse of each DBS train, i.e., the trials were centered on the time cyclic DBS switched off. The subject’s average amount of clean data per condition is provided in Table 2.

**Table 2.** Amount of data per condition, after cleaning

	Mean/SD number of DBS offsets (resting state)	Mean/SD time spent tapping DBS OFF (s)	Mean/SD time spent tapping DBS ON (s)
10 Hz DBS	58.92/4.72	35.42/9.54	56.68/15.03
16 Hz DBS	59.83/5.78	34.60/8.97	55.92/13.49
20 Hz DBS	60.63/4.52	35.42/9.12	56.60/14.16
26 Hz DBS	60.18/4.60	34.69/9.28	56.69/14.28
30 Hz DBS	60.15/4.41	34.37/8.50	56.58/14.15
Baseline (DBS OFF)	49.57/11.18	89.86/21.64	

SD, standard deviation.

#### Time–frequency analysis

We performed time–frequency analyses using the Morlet wavelet transformation in the frequency range from 5 to 60 Hz, with a frequency resolution of 1 Hz. For the spectra shown in Figure 4, we used a wavelet length of 500 ms and plotted the spectra from 5 Hz onwards to provide a clear picture of the localization of the effect in frequency. For the spectra shown in Figure 5*A*, we used shorter wavelets (300 ms). These spectra formed the basis of the analysis of DBS after-effects, which required short wavelets to prevent artifact leakage from the DBS ON into the DBS OFF period. Based on the single-trial wavelet coefficients, we computed power and intertrial coherence (ITC) for each time–frequency bin. Time–frequency spectra were baseline–corrected by subtracting the average power within the baseline period from each time–frequency bin. Note that the baseline value was frequency specific.

#### Sensors of interest

**Figure 3.**
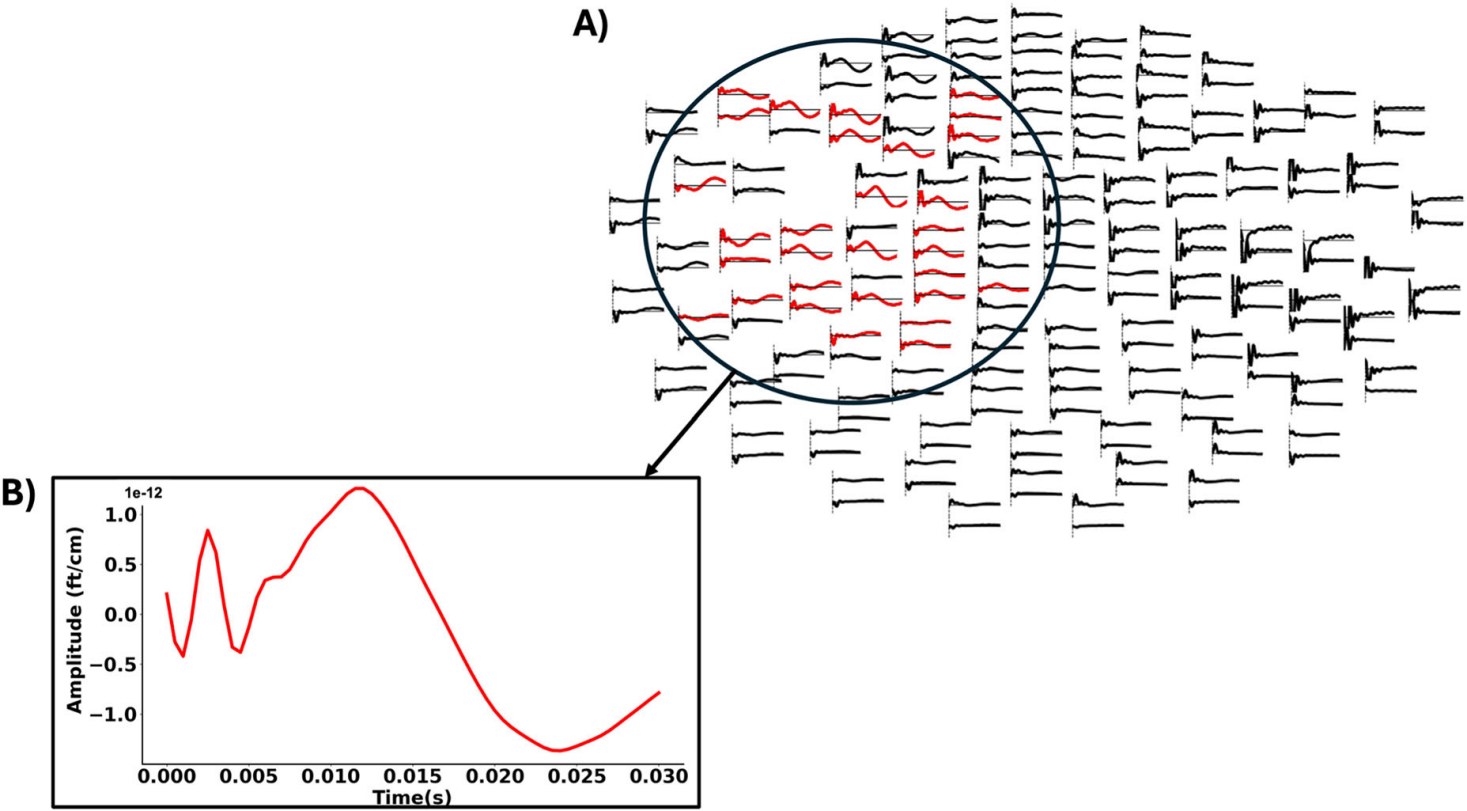
Individual region of interest for one subject. ***A***, The region of interest consisted of magnetoencephalography sensors that showed a clear evoked field in response to the DBS pulses (red). The selected sensors were located in the frontal and sensorimotor regions of the left hemisphere, ipsilateral to stimulation. ***B***, Magnetic field evoked by the DBS pulse averaged over selected sensors.

We defined individual regions of interest (ROI) on the sensor level by visually selecting all MEG sensors showing a clear evoked field in response to the DBS pulses. By basing our selection on evoked fields, which were not under study here, we sought to select regions which are modulated by DBS while avoiding selection bias. The selected sensors were located in frontal and sensorimotor regions of the left hemisphere, i.e., ipsilateral to stimulation, in all cases. On average, we selected 25.93 sensors per participant (SD = 12.84; Fig. 3).

#### Lateralization

Our spectral analyses focused on DBS-induced changes in the lateralization of cortical power, based on the assumption that DBS will modulate cortical oscillations in the stimulated hemisphere more strongly than in the nonstimulated hemisphere. To assess the hemispheric balance of cortical power, we compared the stimulated versus the nonstimulated hemisphere by means of the lateralization index (LI):

$$\mathrm{LI}\;=\;\frac{\left(\mathrm{Left}-\mathrm{Right}\right)}{\left(\mathrm{Left}+\mathrm{Right}\right)},$$

where Left represents band-limited power averaged over the left hemispheric sensors of interest and Right represents the corresponding value for the right hemisphere. The right hemispheric sensors were obtained by mirroring the left ROI across the posteroanterior midline of the sensor array. Positive LI values indicate a left–hemispheric dominance.

### Resting-state lateralization

#### After-effects

We extracted the post–stimulation, time domain data (5–160 ms after the last pulse in a train) and computed the LI to assess the after-effects of DBS in the resting state. The 5 ms margin served to avoid the inclusion of DBS artifacts. The after-effect LI was computed per frequency in the range from 5 to 60 Hz, with a frequency resolution of 6.45 Hz.

#### Baseline

To obtain a resting-state baseline LI used for normalizing the after-effect LI, we processed the baseline (DBS OFF) recording the same way we processed the DBS ON data and computed the LI as above. Because trials could not be centered on the time cyclic DBS switched off, we cut the data into consecutive epochs instead.

#### Tapping lateralization

Tapping lateralization quantifies the lateralization of beta power during finger tapping, computed for cyclic DBS OFF and cyclic DBS ON. To minimize the impact of artifacts in DBS ON, we chose a narrower definition of the beta-band here, focusing on the band with the strongest DBS after-effect (14–18 Hz). For this analysis, we excluded the 16 Hz DBS condition because the stimulation artifact affected the frequency band of interest.

#### Statistical analyses

To investigate short-lived increases of beta power outlasting the stimulation trains, we assessed power differences between the immediate poststimulation phase and mean power in the entire stimulation pause (0.155–2.845) for each stimulation frequency. Therefore, we applied nonparametric, cluster-level, paired *t* tests for spatiotemporal data as implemented in the MNE-Python library (Gramfort et al., 2014) to power in the frequency range from 10 to 23  Hz and three different time windows after DBS cessation (Time 0): T1, 0.155–0.31 s; T2, 0.31–0.465 s; and T3, 0.465–0.62 s. The mean power in each window was compared to the mean of the entire pause. Significant clusters were identified using a permutation test controlling for multiple comparisons in sensor space. The test encompassed all possible permutations. To control for the conduction of several permutation tests, we applied false discovery rate (FDR) correction using the Benjamini–Hochberg method for independent correlated tests and retained clusters that remained significant at a corrected *α* level of 0.05.

In addition, we investigated possible differences in hemispheric power lateralization (LI) between the immediate poststimulation phase (0.005–0.16 s) and the baseline (DBS OFF) recording. This was done by subtracting the baseline LI from the after-effect LI, yielding the normalized LI, and testing whether the normalized LI was different from zero by means of a one-sample permutation *t* test as implemented in the MNE-Python library (Gramfort et al., 2014). This test was performed per frequency to assess the frequency-specificity of the effect, with the “tmax” method controlling for multiple comparisons.

While the cluster permutation tests described above allow for comparisons across all sensors and frequencies, they are simple condition contrasts. For a more comprehensive assessment, we conducted a linear mixed-effects (LME) analysis using the lme4package (version 1.1.26; Bates et al., 2015) in R (version 4.0.4). This model can account for interindividual variance introduced by participants, minimizing the potential for false positives (Yarkoni, 2022).

In the LME analysis, we tested whether the lateralization of cortical beta power differed across the five DBS frequencies. This model included the categorial fixed-effects factor “DBS Frequency” with the five factor levels (10, 16, 20, 26, and 30 Hz) as predictors for the normalized LI. The model further included “Participants” as a random-effects factor. We estimated the model parameters using a restricted maximum likelihood approach (Luke, 2017), and the *α* level for significance was set at 0.05. Degrees of freedom and *p*-values were computed using the Satterthwaite approximation, implemented in the lmerTest package (version 3.1; Kuznetsova et al., 2017).

A similar analysis was used to investigate the effects of beta-band DBS on finger tapping. The model included the categorical fixed-effects factors, “DBS” [ON (+0.5) and OFF (−0.5)] and “DBS Frequency” with five factor levels (10, 16, 20, 26, and 30 Hz), along with their interaction, as predictors for the tap rate. The model further included “Participants” as a random-effects factor. Degrees of freedom and *p*-values were estimated based on the likelihood ratio test method based on Type III sums of squares and the *α* level for significance was set at 0.05. Because tapping, when expressed as an integer tap per minute, represents count data, we used a Poisson distribution to model finger taps. This generalized linear mixed-effects analysis was conducted with the afex package (version 1.1-1; Singmann et al., 2015). For further statistical data analyses and data visualization, including violin plots and Pearson's correlation coefficients, we used the R Stats and R Base packages, as well as ggplot2 (version 3.3.3; Wickham, 2016).

## Results

### Effects of beta-band DBS on resting-state oscillations

**Figure 4.**
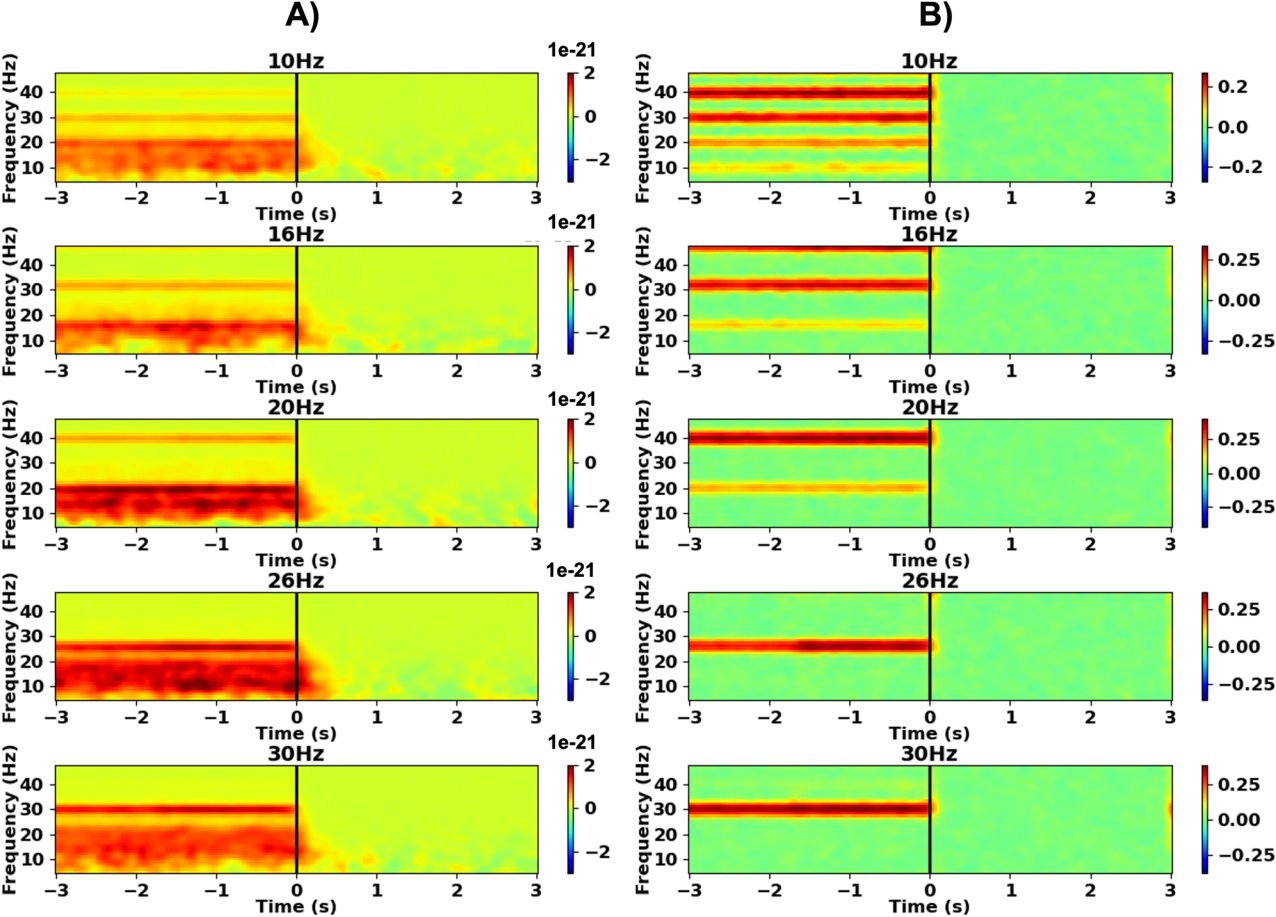
Beta-band deep brain stimulation-induced power changes, not phase alignment. The time–frequency spectra depict resting-state power (***A***) and intertrial coherence (***B***) averaged over left sensorimotor sensors of interest and subjects. DBS was switched off at time 0 (vertical black line). The power difference to the stimulation pause mean (0.275–2.725 s) is color-coded. The DBS artifact topography is shown in Extended Data Figure 4-1.

In the resting state, beta-band DBS increased cortical power between 8 and 23 Hz (Fig. 4*A*). Importantly, this effect persisted for ∼400 ms after cyclic beta-band DBS switched off and differed spectrally and topographically from DBS artifacts (see Extended Data Fig. 4-1 for the artifact topography), indicating a physiological origin. Notably, we did not observe any after-effects in intertrial coherence, indicating that beta-band DBS did not lead to phase alignment of oscillations with respect to the DBS pulse train (Fig. 4*B*).

10.1523/JNEUROSCI.1366-24.2024.f4-1 Figure 4-1 **Deep brain stimulation artifact.** Group-average time-frequency spectra for right parietal sensors depicting the deep brain stimulation (DBS) artifact during 26  Hz and 30  Hz stimulation. DBS was switched off at time 0. Power change relative to the mean over the stimulation pause (0.155-2.845  s) is color-coded. The black rectangles mark the time-frequency selection for averaging power in the topographical plots (time windows: -3.00-0.00  s; frequency range: DBS frequency). Spectrally, the artifact varies as a function of stimulation frequency. Spatially, the artifact is governed by the trajectory of the subcutaneous wires connecting electrodes and stimulator (right parietal and right occipital sensors). Download Figure 4-1, TIF file.

**Figure 5.**
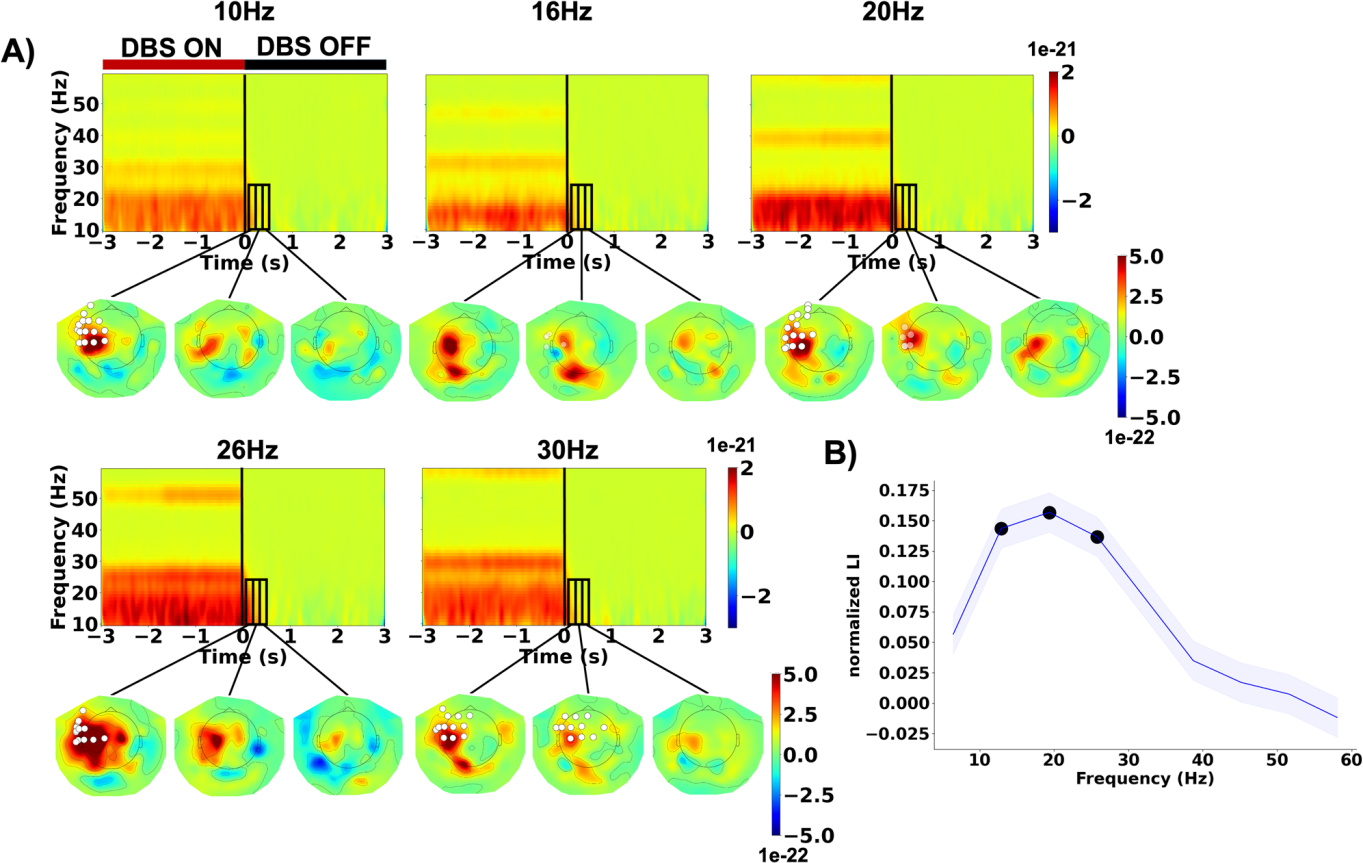
Beta-band deep brain stimulation increased cortical beta power in a spatially and spectrally selective manner. ***A***, Group average time–frequency spectra showing resting-state power for the left sensorimotor channels of interest. Deep brain stimulation was switched off at Time 0. The power difference to the stimulation pause mean (0.155–2.845 s) is color-coded. The black rectangles mark the time–frequency selection for averaging power in the topographical plots (time windows, 0.155–0.31 s; 0.31–0.465 s; 0.465–0.62 s; frequency range, 10–23 Hz). White dots indicate a significant deviation from the stimulation pause mean (0.155–2.845 s) after applying the false discovery rate (FDR) correction. Transparent dots represent deviations that did not meet the criteria for significance after FDR correction. See Extended Data Table 5-1 for cluster statistics. See Extended Data Figure 5-1 for the results of the same analysis applied within the gamma frequency range (32–60 Hz). ***B***, Lateralization of power in the region of interest. The lateralization index (LI) for power 0.005–0.16 s poststimulation was normalized by subtracting the baseline LI and averaged across sessions and participants. The blue shaded area represents the standard error. Positive values indicate stronger left–hemispheric dominance than observed at baseline. The black dots indicate that the normalized LI was significantly different from zero.

The after-effect was most prominent in frontal and sensorimotor regions of the left hemisphere, ipsilateral to stimulation, as indicated by cluster-level permutation tests (Fig. 5*A*; see Extended Data Table 5-1 for cluster statistics). Finally, the poststimulation boost of cortical beta power in the stimulated hemisphere resulted in a shift of hemispheric beta power lateralization beyond baseline levels, specifically for beta frequencies (Fig. 5*B*; effect at 12.9–25.8 Hz, *t* ≥ 3.1, *p* ≤ 0.042). This effect occurred for all stimulation frequencies tested here, with no significant main effect of “DBS Frequency” on the normalized after-effect LI (*F*_(4, 42.12)_ = 1.32, *p* = 0.277). We found no significant power changes within the gamma frequency range (32–60 Hz, Extended Data Fig. 5-1).

10.1523/JNEUROSCI.1366-24.2024.t5-1 Table 5-1 **Clusters identified with non-parametric cluster-level paired t-tests.** Time windows after DBS cessation (time 0): T1: 0.155-0.31  s, T2: 0.31-0.465  s, T3: 0.465-0.62  s. No significant clusters were identified for T3. Download Table 5-1, DOCX file.

10.1523/JNEUROSCI.1366-24.2024.f5-1 Figure 5-1 **Beta-band deep brain stimulation did not increase cortical gamma power.** Group-average time-frequency spectra for the left sensorimotor channels of interest, in resting-state. Deep brain stimulation was switched off at time 0. The power difference to the stimulation pause mean (0.155-2.845  s) is color-coded. The black rectangles mark the time-frequency selection for averaging power in the topographical plots (time windows: 0.155-0.31  s; 0.31-0.465  s; 0.465-0.62  s; frequency range: 32-60  Hz). Transparent dots represent effects found in permutation testing. None of these met the criteria for significance after False Discovery Rate (FDR) correction. Download Figure 5-1, TIF file.

### Effects of beta-band DBS on finger tapping

**Figure 6.**
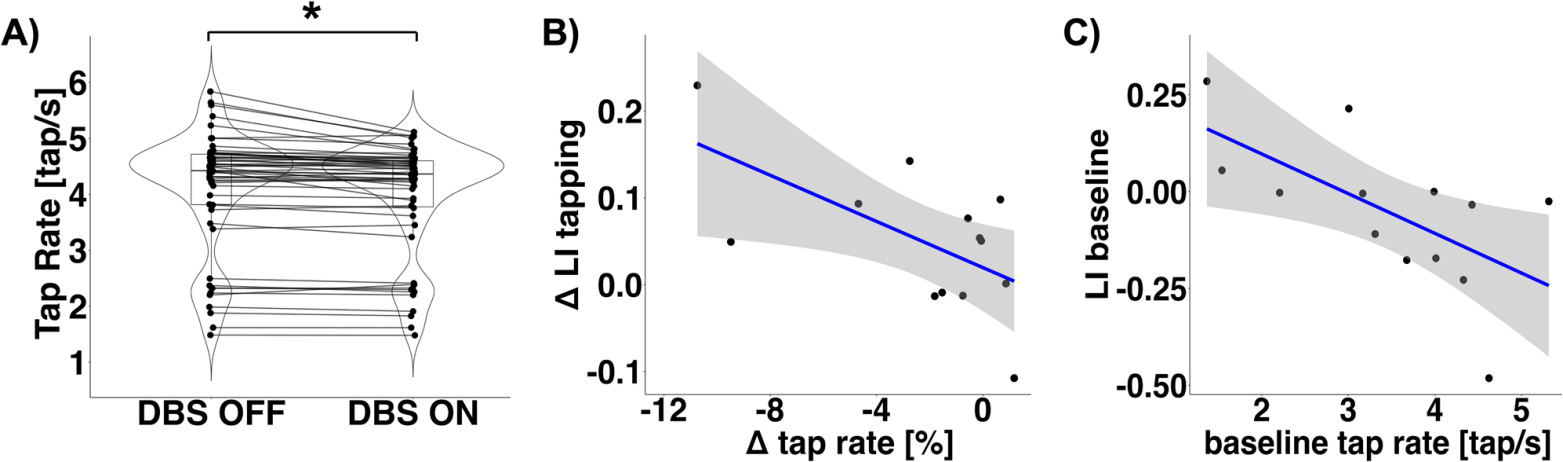
Beta-band deep brain stimulation slowed down finger tapping and stronger modulation of beta power was associated with stronger slowing. ***A***, Distribution of the tap rate for the pauses in between stimulation trains (DBS OFF) and when beta-band DBS was switched on (DBS ON). The embedded box-and-whisker plots represent the 25th and the 75th percentiles of the distributions, respectively. The vertical lines indicate median values. Each dot represents one measurement (14 participants, 2–5 stimulation frequencies per participant). *p < 0.05. ***B***, Correlation between DBS-induced change of tap rate and simultaneously induced change of beta power lateralization, as quantified by the lateralization index (LI). The *y*-axis denotes the difference in LI between DBS OFF and DBS ON during finger tapping. Positive values on the *y*-axis indicate that DBS increased cortical beta power in the left (stimulated) hemisphere relative to the right (nonstimulated) hemisphere. Negative values on the *x*-axis indicate that tapping was slowed down by DBS. Each dot represents one participant (session average). The gray shaded area represents the respective 95% confidence interval. ***C***, Correlation between tap rate and beta power lateralization in the baseline (DBS OFF) recording.

Consistent with previous studies (Fogelson et al., 2005; Chen et al., 2007; Eusebio et al., 2008; Pogosyan et al., 2009; Joundi et al., 2012), we observed a slight slowing of movement when beta-band DBS was switched on, as compared with the pauses in between stimulation trains (main effect of “DBS” on tap rate:*χ*^2^_(1)_ = 4.91, *p* = 0.027, Fig. 6*A*). Neither the main effect of “DBS Frequency” (*χ*^2^_(4)_ = 6.61, *p* = 0.158) nor the interaction of both predictors was significant (*χ*^2^_(4)_ = 0.41, *p* = 0.982), suggesting that all tested DBS frequencies modulated tapping in a similar fashion.

### Correlations between electrophysiology and behavior

We observed a correlation between the electrophysiological and the behavioral effects of beta-band DBS. A stronger DBS-induced shift of beta power toward the stimulated hemisphere was associated with stronger DBS-induced slowing (*r* = −0.63, *p* = 0.021; Fig. 6*B*). Notably, the negative correlation between hemispheric lateralization of beta power and the tap rate was observable in the baseline (DBS OFF) condition, too. These findings indicate that, by increasing beta power lateralization, DBS accentuated an existing antikinetic pattern, characterized by a relatively high level of beta power in the hemisphere contralateral to movement (*r* = −0.61, *p* = 0.028; Fig. 6*C*).

## Discussion

In the present study, PD patients underwent MEG recordings while receiving STN beta-band DBS, both at rest and during right-hand finger tapping. Beta-band DBS slowed down finger tapping and increased motor cortical alpha/beta power in the stimulated hemisphere relative to the unstimulated hemisphere. This neurophysiological effect did not vary as a function of stimulation frequency and correlated with tap rate reduction, suggesting that subthalamic beta-band DBS leads to motor slowing by boosting, not entraining, beta oscillations in motor cortex.

### Cortical beta power, hemispheric beta lateralization, and akinesia

In the baseline recording (DBS OFF), we observed a negative correlation between the strength of hemispheric beta power lateralization and the tap rate, indicating that a relatively high level of beta power in the hemisphere contralateral to the moving hand is hampering movement. This notion aligns with previous research. Pollok et al. (2012), for example, observed a correlation between motor impairment and beta power in the motor cortex contralateral, but not ipsilateral, to movement. Heinrichs-Graham et al. (2017) studied beta power suppression preceding movement and found that its lateralization reflected the lateralization of symptoms. In conjunction, these studies demonstrate that the hemispheric balance of beta power is relevant to motor impairment. Note, however, that other studies found an inverse relationship between the absolute level of resting state, cortical beta power, and symptom severity (Cao et al., 2020), emphasizing the need to differentiate between beta power lateralization and the absolute level of cortical beta power.

Given the negative correlation between resting-state beta power lateralization and tapping speed at baseline, we consider a strong lateralization a pathological pattern, which we found to be accentuated by beta-band DBS. At the same time, beta-band DBS slowed down finger tapping compared with baseline, consistent with previous studies reporting a slowing of movement (Fogelson et al., 2005; Chen et al., 2007; Eusebio et al., 2008; Pogosyan et al., 2009; Joundi et al., 2012) or a worsening of motor symptoms (Timmermann et al., 2004) by electrical stimulation at frequencies <30 Hz. Importantly, the neuromodulatory and behavioral effects were linearly related: the stronger the accentuation of lateralization, the stronger the slowing induced by beta-band DBS. This relation is most likely not based on artifacts caused by movement or DBS, which would tend to distort any neurobehavioral correlation.

While not a proof of causality (Neumann et al., 2016), the observed correlation is consistent with the hypothesis that beta-band DBS causes motor slowing by amplifying cortical beta oscillations, similar to direct electrical stimulation of the motor cortex (Pogosyan et al., 2009; Joundi et al., 2012). If this is correct, diminishing cortical beta activity should go along with motor improvement. Indeed, high-frequency DBS, which improves motor symptoms, was found to reduce motor cortical beta power in some (Abbasi et al., 2018; Luoma et al., 2018) but not all studies (Cao et al., 2015; 2017). Interestingly, Hall et al. (2014) reported analogous effects resulting from pharmacological intervention. They applied the GABA_A_ receptor agonist zolpidem in PD patients and found the exact opposite of what we report for beta-band DBS. The drug improved rather than worsened movement and diminished rather than accentuated the hemispheric power difference (Hall et al., 2014). In their study, the equalizing effect of zolpidem correlated with motor improvement, whereas in our study, the diverging effect of beta-band DBS correlated with motor slowing. That being said, we acknowledge that both the motor slowing and the increase of cortical beta power might be caused by another, unknown mechanism.

### Entrainment

The term entrainment refers to the process of imposing a new rhythm on neural oscillators by means of rhythmic sensory (Williams, 2001; Spaak et al., 2014) or electrical stimulation (Thut et al., 2011; Helfrich et al., 2014)*.* Investigating the latter is challenging due to the artifacts produced by electrical stimulation. Here, we largely avoided those artifacts by focusing on the after-effects of beta-band DBS. The modulation of cortical beta lateralization outlasted DBS by a few hundred milliseconds. Similar after-effects have been demonstrated on the level of the subthalamic nucleus, where 130 Hz DBS produces a suppression of local beta power outlasting stimulation (Kühn et al., 2008; Wiest et al., 2020). The presence of after-effects rules out confounds introduced by electromagnetic DBS artifacts because these disappear the moment DBS is switched off. Moreover, the topography of the neuromodulatory effect did not match the artifact topography, which is governed by the path of the subcutaneous wires connecting electrodes and stimulator (Kandemir et al., 2020). Lastly, the peak frequency of the effect remained stable at ∼15 Hz even though the stimulation frequency varied across conditions. The latter observation is not only a strong argument against DBS artifact-related confounds but also against entrainment, the effects of which should vary as a function of stimulation frequency. Specifically, the amplitude modulations should be strongest when the stimulation frequency is close to the resonance frequency of the system (Notbohm et al., 2016; Vosskuhl et al., 2018). In addition, entrainment is characterized by phase alignment between neural oscillations and rhythmic stimulation (Hanslmayr et al., 2019). Here, we tested phase alignment by computing intertrial coherence, a measure of phase consistency across trials. In contrast to power, intertrial coherence was not elevated immediately after DBS was turned off. Hence, we conclude that the neurophysiological effects of beta-band DBS described here are not based on entrainment but represent a remote-induced response.

The location of this response, namely, sensorimotor areas ipsilateral to the stimulated STN, is plausible based on the known structural and functional connectivity of the STN. Resting-state, beta-band coherence between STN and cortex, for example, peaks in the ipsilateral sensorimotor cortex (Hirschmann et al., 2011; Litvak et al., 2011; van Wijk et al., 2016). This functional connection is believed to rely on the hyperdirect pathway (Oswal et al., 2021; Steina et al., 2024), a monosynaptic connection from the motor cortex to the STN formed by axon collaterals of the internal capsule (Nambu et al., 2002; Aron et al., 2007; Chen et al., 2020). Whether the observed neuromodulatory effects are indeed based on the hyperdirect pathway or other connections like the indirect pathway cannot be inferred from the data at hand.

Interestingly, remote-induced responses have also been reported for high-frequency DBS, but in the gamma range rather than in the beta-band. Wiest et al. (2021) observed finely tuned gamma oscillations emerging in response to high-frequency DBS in PD patients off medication, coherent across the STN and cortex. Similar to the beta-band response described here, these oscillations outlasted stimulation, albeit for several seconds rather than a few hundred milliseconds, and did not change with stimulation frequency. Accordingly, these effects were not attributed to entrainment. In the medication on state, finely tuned gamma oscillations are exaggerated and associated with dyskinesia (Swann et al., 2016; 2018; Olaru et al., 2024). In this situation, there are some hints of entrainment: when DBS is switched on in patients with dyskinesia, the peak of finely tuned gamma oscillations shifts to half the stimulation frequency (Swann et al., 2016; Oehrn et al., 2024). Furthermore, the reported correlation between an estimate of subthalamic power at stimulation frequency and cortical gamma power might be viewed as a form of entrainment, although not necessarily in the sense of phase alignment (Muthuraman et al., 2020). Irrespective of the findings made for gamma oscillations, our results suggest that beta oscillations may be amplified by beta-band DBS, but not entrained, not even in medication ON.

### Clinical and neuroscientific relevance

We report the possibility of amplifying cortical beta oscillations experimentally through beta-band DBS. This capacity opens new possibilities for clinical neuroscience, in particular with respect to the discussion about the causal role of beta oscillations. Are they really the “bad boy of parkinsonism” (Eusebio and Brown, 2009) causing akinesia and rigidity or merely an epiphenomenon? While the former is hard to prove, even with neuromodulation, it can be disproved with the method at hand. A strong boost of beta activity without any sign of motor slowing would be incompatible with causal interactions. Here, however, we found that both go hand in hand, supporting the notion of an “anitkinetic” nature. Future studies may transfer the approach to other domains involving cortical beta oscillations, like decision-making (Haegens et al., 2011; Chen and Kwak, 2022), working memory (Schmidt et al., 2019), or language processing (Weiss and Mueller, 2012), for example.

### Limitations

This study lacked subcortical measurements. In consequence, we cannot say whether the observed slowing results from the amplification of cortical beta oscillations or subcortical DBS effects. Furthermore, the motor slowing achieved by beta-band DBS was rather small (mean difference = 6.67 taps per minute = 0.11 taps per second, Cohen’s *d* = 0.57), consistent with previous reports (Fogelson et al., 2005; Chen et al., 2007; Eusebio et al., 2008; Pogosyan et al., 2009; Joundi et al., 2012).

### Conclusion

Beta-band DBS amplifies an “antikinetic” cortical pattern, marked by heightened beta power in the hemisphere contralateral to movement, and slows down movement. These effects are correlated and consistent with a causal role of beta oscillations in motor control.
