# Supplementary figures and images for: Subthalamic Nucleus Deep Brain Stimulation in the Beta Frequency Range Boosts Cortical Beta Oscillations and Slows Down Movement
Source: J Neurosci. 2025 Jan 9;45(9):e1366242024. doi: 10.1523/JNEUROSCI.1366-24.2024 (PMC11867002; doi:10.1523/JNEUROSCI.1366-24.2024)

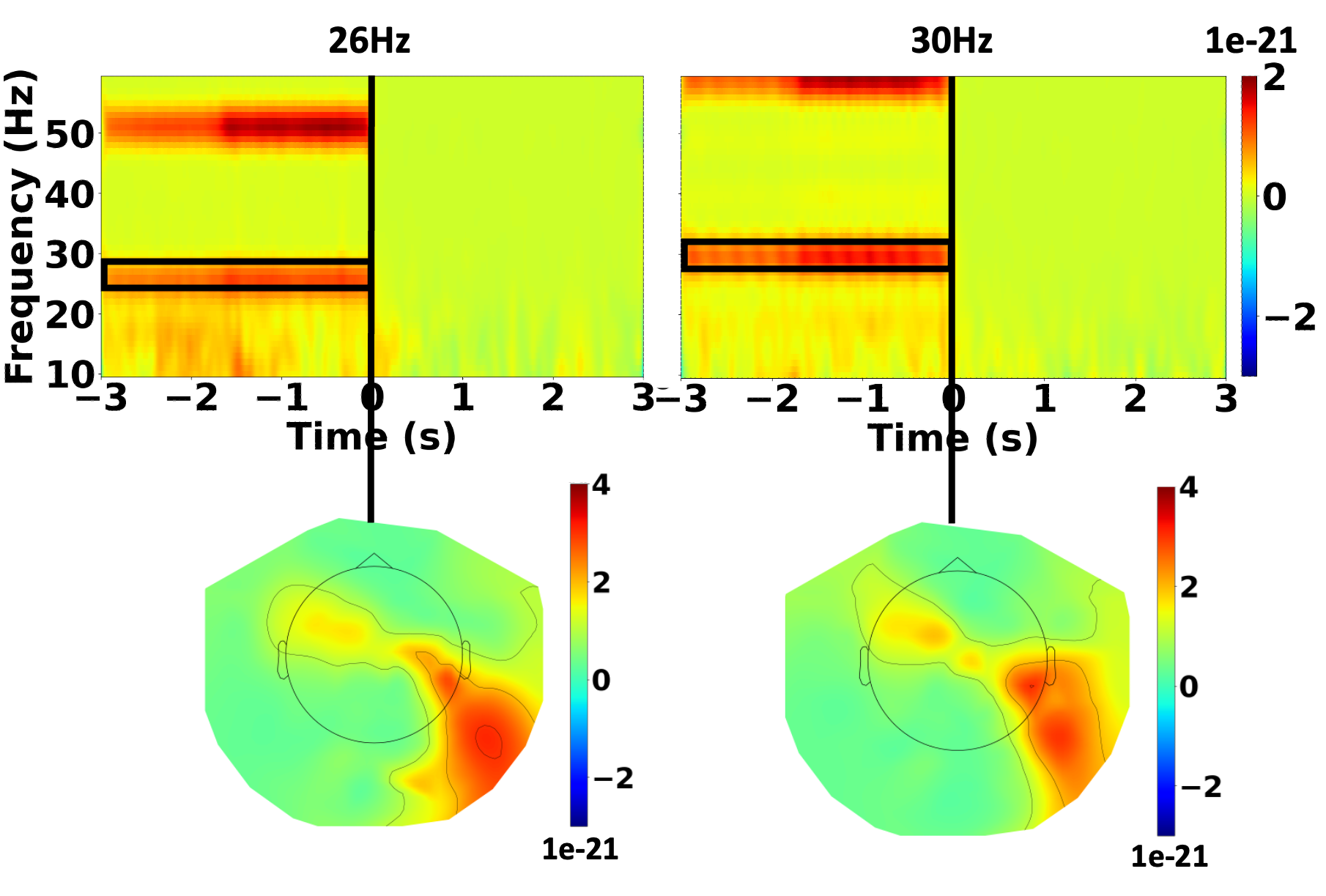

Supplement: Figure 4-1 — Deep brain stimulation artifact. Group-average time-frequency spectra for right parietal sensors depicting the deep brain stimulation (DBS) artifact during 26 Hz and 30 Hz stimulation. DBS was switched off at time 0. Power change relative to the mean over the stimulation pause (0.155-2.845 s) is color-coded. The black rectangles mark the time-frequency selection for averaging power in the topographical plots (time windows: -3.00-0.00 s; frequency range: DBS frequency). Spectrally, the artifact varies as a function of stimulation frequency. Spatially, the artifact is governed by the trajectory of the subcutaneous wires connecting electrodes and stimulator (right parietal and right occipital sensors). Download Figure 4-1, TIF file. [file jneuro-45-e1366242024-s003.tif]

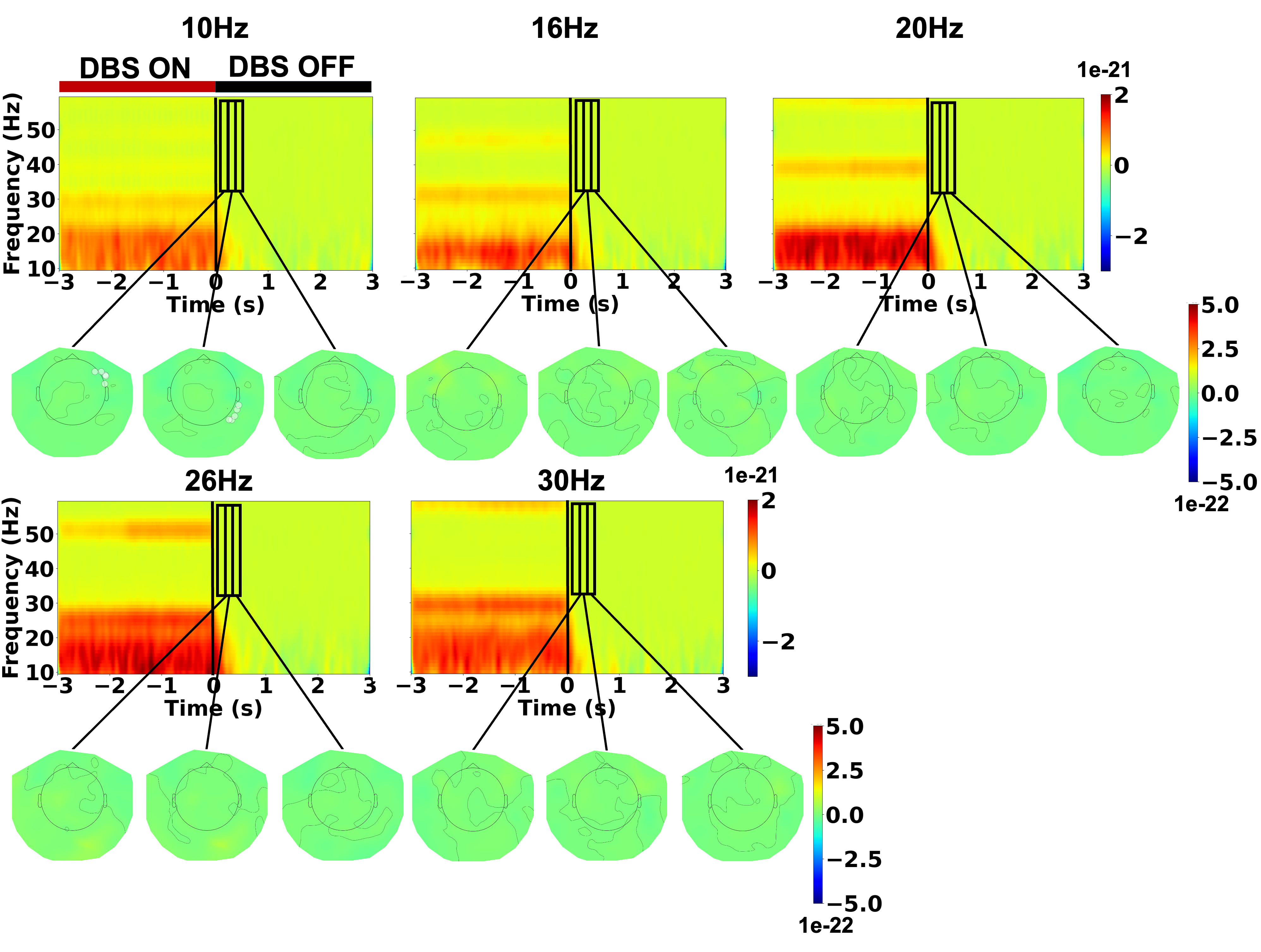

Supplement: Figure 5-1 — Beta-band deep brain stimulation did not increase cortical gamma power. Group-average time-frequency spectra for the left sensorimotor channels of interest, in resting-state. Deep brain stimulation was switched off at time 0. The power difference to the stimulation pause mean (0.155-2.845 s) is color-coded. The black rectangles mark the time-frequency selection for averaging power in the topographical plots (time windows: 0.155-0.31 s; 0.31-0.465 s; 0.465-0.62 s; frequency range: 32-60 Hz). Transparent dots represent effects found in permutation testing. None of these met the criteria for significance after False Discovery Rate (FDR) correction. Download Figure 5-1, TIF file. [file jneuro-45-e1366242024-s004.tif]
