# Supplementary material for: Subthalamic Nucleus Deep Brain Stimulation in the Beta Frequency Range Boosts Cortical Beta Oscillations and Slows Down Movement
Source: J Neurosci. 2025 Jan 9;45(9):e1366242024. doi: 10.1523/JNEUROSCI.1366-24.2024 (PMC11867002; doi:10.1523/JNEUROSCI.1366-24.2024)
Supplement: Table 5-1 — Clusters identified with non-parametric cluster-level paired t-tests. Time windows after DBS cessation (time 0): T1: 0.155-0.31 s, T2: 0.31-0.465 s, T3: 0.465-0.62 s. No significant clusters were identified for T3. Download Table 5-1, DOCX file. [file jneuro-45-e1366242024-s002.docx]

Table 5-1. Clusters identified with non-parametric cluster-level paired t-tests.

| Stimulation Frequency | Time window | t_clustermean_ | p-value | p-value (FDR corrected) |
| --- | --- | --- | --- | --- |
| 10Hz |  |  |  |  |
|  | T1 | 2.43 | 0.008 | 0.023 |
| 16Hz |  |  |  |  |
|  | T2 | 2.81 | 0.037 | 0.079 |
| 20Hz |  |  |  |  |
|  | T1 | 2.88 | 0.005 | 0.018 |
|  | T2 | 3.13 | 0.034 | 0.079 |
| 26Hz |  |  |  |  |
|  | T1 | 2.69 | < .001 | 0.015 |
| 30Hz |  |  |  |  |
|  | T1 | 2.85 | 0.003 | 0.015 |
|  | T2 | 2.61 | 0.003 | 0.015 |

Time windows after DBS cessation (time 0): T1: 0.155-0.31s, T2: 0.31-0.465s, T3: 0.465-0.62s. No significant clusters were identified for T3.
